# Application of Yang homotopy perturbation transform approach for solving multi-dimensional diffusion problems with time-fractional derivatives

**DOI:** 10.1038/s41598-023-49029-w

**Published:** 2023-12-09

**Authors:** Jinxing Liu, Muhammad Nadeem, Loredana Florentina Iambor

**Affiliations:** 1 Faculty of Science, Yibin University, Yibin, 644000 China; 2 School of Mathematics and Statistics, Qujing Normal University, Qujing, 655011 China; 3 Department of Mathematics and Computer Science, University of Oradea, 1 University Street, 410087 Oradea, Romania

**Keywords:** Applied mathematics, Fluid dynamics

## Abstract

In this paper, we aim to present a powerful approach for the approximate results of multi-dimensional diffusion problems with time-fractional derivatives. The fractional order is considered in the view of the Caputo fractional derivative. In this analysis, we develop the idea of the Yang homotopy perturbation transform method (YHPTM), which is the combination of the Yang transform (YT) and the homotopy perturbation method (HPM). This robust scheme generates the solution in a series form that converges to the exact results after a few iterations. We show the graphical visuals in two-dimensional and three-dimensional to provide the accuracy of our developed scheme. Furthermore, we compute the graphical error to demonstrate the close-form analytical solution in the comparison of the exact solution. The obtained findings are promising and suitable for the solution of multi-dimensional diffusion problems with time-fractional derivatives. The main advantage is that our developed scheme does not require assumptions or restrictions on variables that ruin the actual problem. This scheme plays a significant role in finding the solution and overcoming the restriction of variables that may cause difficulty in modeling the problem.

## Introduction

The study of fractional calculus is becoming more interesting in various branches of mathematical problems including integral and derivatives of fractional order. The phenomena of fractional order problems have a great attraction in other branches of science and engineering such as as astronomy, optical fiber, biomechanics, chemical reactions, heat transform, and fluid flows^1,2^. In recent years, numerous researchers have introduced the analytical and numerical approaches to obtain their approximate solutions. Malan and Lewis^3^ utilized edge-based finite volume method to model heat and mass transfer in heterogeneous porous materials. Arafa and Hagag^4^ presented *q*-Homotopy analysis transform method for the analytic solution of fractional coupled Ramani problem. El-Sayed et al.^5^ developed the idea of Adomian’s decomposition method for the approximate solution of the reaction-diffusion model of fractional order. It is still challengeable task to obtain the exact solution of these fractional problems. Most of the fractional system do not have the exact solutions due to the difficulty of fractional order. To investigate their approximate solutions, various authors presented their schemes that obtain the results very close to the exact solution such as Fractional Temimi–Ansari method^6^, Differential transform scheme^7^, Haar wavelet operational matrix^8^, Natural transform^9^, Sumudu residual power series method^10^, Finite difference approach^11^, High-order finite element scheme^12^, Local fractional Sumudu transform^13^, Sub-equation method^14^.

This work is concerned with the time fractional multi-dimensional diffusion equation^15,16^:

1 $$\begin{aligned} D^{\alpha }_{\tau }\vartheta =\nabla . (D(\vartheta (r,\tau ),r)\nabla \vartheta (r,\tau )), \qquad 0<\alpha \le 1 \end{aligned}$$

where $D^{\alpha }_{\tau }=\dfrac{\partial ^{\alpha }}{\partial \tau ^{\alpha }}$ stands for the Caputo fractional derivative, $\vartheta (r,\tau )$ and $D\vartheta (r,\tau )$ represent the density of the diffusing material and the diffusion coefficient for $\vartheta$ at the point $r = (x, y, z)$ and time $\tau$ respectively. If the diffusion coefficient is free from density (i.e. $D\vartheta (r,\tau )=\sigma ^{2}$ is a constant), then problem (1) tends to the fractional order multi-dimensional heat equation, such that $D^{\alpha }_{\tau }\vartheta =\sigma ^{2}\nabla ^{2}\vartheta$. In case of $\alpha =1$, the problem (1) reduces to the classical multi-dimensional diffusion equation.

Recently, Yang^17^ proposed the idea of Yang transform for the first time and showed that this scheme is straightforward for deriving the results of a steady heat transfer equation. The idea of homotopy perturbation method (HPM) was constructed by He in 2004 and showed that this scheme is suitable for different types of problems^18^. Later, many researchers extend this study and combined HPM to obtain the approximate solution of some more fractional differential problems. Liu^19^ et al. combined Yang transform with HPM to derive the analytical results of time-fractional Klein–Gordon problems. Yasmin^20^ combined Yang transform with the Adomian decomposition approach to present the analysis of the Whitham–Broer–Kaup problem with time-fractional order. The Yang transform with HPM performed excellent results in finding a solution of fractional order KdV and Burger problem^21^. The study of HPM^22^ has becoming more and more interesting and numerous researchers have showed the combination of HPM with an other operator produces faster rate of convergence^23,24^. Akbarzade and Langari^25^ showed that HPM is more reliable tool than variational iteration scheme in finding the approximate results of three dimensional heat problems. Kumar et al.^16^ applied the modification of HPM whereas Prakash and Kumar^26^ suggested the application of fractional variational iteration scheme to present the analytical view of multi-dimensional diffusion problems. Researchers showed that combination of these transformation with the HPM provide the excellent results than the traditional HPM. Since various analytical and numerical schemes are presented by experts in the literature. In the most of schemes, authors have faced some difficulties and limitations due to the heavy calculations in the iteration series. The use of integration in variational iteration scheme and convolution theorem Laplace transform make the solution complicated and may occur some assumption and restrictions on variables that is the main drawback of these schemes^27,28^. To overcome, this drawback, we propose the idea of YHPTM for the approximate solution of multi-dimensional diffusion problems with time-fractional derivatives.

In this work, we combine the YT with HPM to develop a novel scheme that is expressed by YHPTM. We consider a few problems to test the accuracy and performance of this proposed scheme. We note that our developed scheme produces results very close to the exact results after a few iterations and some graphical visuals are also provided to show its performance with graphical errors. We begin this article as; we present the idea of Yang transform in “Concept of Yang transform” including its definitions. We develop the idea of YHPTM for the solution of fractional problems and provide its convergence analysis in “Formulation of YHPTM” and “Convergence and error analysis” respectively. In “Applications”, we illustrate some examples to test the compactness and authenticity of our proposed scheme. We conclude our study in the last section “Conclusion”.

## Concept of Yang transform

In this segment, we define the concept of YT with its basic properties.

### Definition 2.1

The Caputo fractional derivative is defined as^29,30^

$$\begin{aligned} D^{\alpha }_{\tau }\vartheta (\Im ,\tau )=\frac{1}{\Gamma (k-\alpha )}\int _{0}^{\tau }(\tau -q)^{k-\alpha -1}\vartheta ^{k}(\Im ,q)\ dq, \qquad k-1 < \alpha \le k. \end{aligned}$$

### Definition 2.2

The YT is stated as^17,19^

$$\begin{aligned} Y[\vartheta (\tau )]=R(\xi )=\int _{0}^{\infty } e^{-\dfrac{\tau }{\xi }} \vartheta (\tau ) d \tau {,} \end{aligned}$$

whereas ${\mathcal {Y}}^{-1}[R(\xi )]=\vartheta (\tau )$ is known as the inverse of YT.

### Definition 2.3

The YT of a fractional derivative is given as^17,19^

$$\begin{aligned} Y[\vartheta ^{\alpha }(\tau )]=\frac{R(\xi )}{\xi ^{\alpha }}-\sum _{k=0}^{n-1}\frac{\vartheta ^{k}(0)}{\xi ^{\alpha -k-1}},\qquad n-1 < \alpha \le n. \end{aligned}$$

### Proposition

*The differential properties of YT for a function* $\vartheta (\tau )$ *are defined as*^19^

$$\begin{aligned} \begin{aligned} Y[\vartheta '(\tau )]&=\frac{R(\xi )}{\xi }-\vartheta (0),\\ Y[\vartheta ''(\tau )]&=\frac{R(\xi )}{\xi ^{2}}-\frac{\vartheta (0)}{\xi }-\vartheta '(0). \end{aligned} \end{aligned}$$

## Formulation of YHPTM

In this section, we construct the idea of YHPTM which is used to derive the approximate results of multi-dimensional diffusion problems with time-fractional derivatives. This scheme does not require the restriction of variables and any hypothesis. Let’s assume the following differential problem of time-fractional order as

2 $$\begin{aligned} D^{\alpha }_{\tau }\vartheta (\Im ,\tau )=L_{1}\vartheta (\Im ,\tau )+L_{2}\vartheta (\Im ,\tau )+h(\Im ,\tau ), \end{aligned}$$

with initial condition

3 $$\begin{aligned} \vartheta (\Im ,0)=k(\Im ). \end{aligned}$$

Operating YT on Eq. (2) such as

$$\begin{aligned} Y[D^{\alpha }_{\tau }\vartheta (\Im ,\tau )]=Y[L_{1}\vartheta (\Im ,\tau )+L_{2}\vartheta (\Im ,\tau )+h(\Im ,\tau )]. \end{aligned}$$

This implies

$$\begin{aligned} \frac{1}{\xi ^{\alpha }}\Big [R(\xi )-\xi \vartheta (0)\Big ]=Y[L_{1}\vartheta (\Im ,\tau )+L_{2}\vartheta (\Im ,\tau )+h(\Im ,\tau )]. \end{aligned}$$

Hence $R(\xi )$ is evaluated such as

4 $$\begin{aligned} R[\xi ]=\xi \vartheta (0)+\xi ^{\alpha } Y\Big [L_{1}\vartheta (\Im ,\tau )+L_{2}\vartheta (\Im ,\tau )+h(\Im ,\tau )\Big ]. \end{aligned}$$

Operating inverse YT on Eq. (4), it yields

5 $$\begin{aligned} \vartheta (\Im ,\tau )=G(\Im ,\tau )+Y^{-1}\Bigg [\xi ^{\alpha } \wp \Big \{L_{1}\vartheta (\Im ,\tau )+L_{2}\vartheta (\Im ,\tau )\Big \}\Bigg ], \end{aligned}$$

where

$$\begin{aligned} G(\Im ,\tau )=Y^{-1}\Big [\xi \vartheta (0)+\xi ^{\alpha }Y[h(\Im ,\tau )]\Big ]. \end{aligned}$$

Now, HPM is defined as

6 $$\begin{aligned} \vartheta (\Im ,\tau )=\sum _{i=0}^{\infty }p^{i}\vartheta _{i}(\Im ,\tau ), \end{aligned}$$

and

7 $$\begin{aligned} L_{2}\vartheta (\Im ,\tau )=\sum _{i=0}^{\infty }p^{i}H_{i}(\vartheta ){,} \end{aligned}$$

where $H_{n}$ polynomials are expressed as;

$$\begin{aligned} H_{n}(\vartheta _{0},\vartheta _{1},\ldots , \vartheta _{n})=\frac{1}{n!}\frac{\partial ^{n}}{\partial p^{n}}\Bigg (L_{2}\Big (\sum _{i=0}^{\infty } p^{i}\vartheta _{i}\Big )\Bigg )_{p=0}, \ \ \ \ n=0,1,2,\ldots {.} \end{aligned}$$

Use Eqs. (6) and (7) in Eq. (5), it yields

8 $$\begin{aligned} \sum _{i=0}^{\infty }p^{i}\vartheta _{i}(\Im ,\tau )=G(\Im ,\tau )+Y^{-1}\Bigg [\xi ^{\alpha } Y\Big \{L_{1}\sum _{i=0}^{\infty }p^{i}\vartheta _{i}(\Im ,\tau )+\sum _{i=0}^{\infty }p^{i}H_{i}(\vartheta )\Big \}\Bigg ]. \end{aligned}$$

Comparing the coefficient of *p*, we obtain

$$\begin{aligned} p^{0}&:\vartheta _{0}(\Im ,\tau )=G(\Im ,\tau ),\\ p^{1}&:\vartheta _{1}(\Im ,\tau )=Y^{-1}\Bigg [\xi ^{\alpha } Y\bigg \{\vartheta _{0}(\Im ,\tau )+H_{0}(\vartheta )\bigg \}\Bigg ],\\ p^{2}&:\vartheta _{2}(\Im ,\tau )=Y^{-1}\Bigg [\xi ^{\alpha } Y\bigg \{\vartheta _{1}(\Im ,\tau )+H_{1}(\vartheta )\bigg \}\Bigg ],\\ p^{3}&:\vartheta _{3}(\Im ,\tau )=Y^{-1}\Bigg [\xi ^{\alpha } Y\bigg \{\vartheta _{2}(\Im ,\tau )+H_{2}(\vartheta )\bigg \}\Bigg ],\\&\vdots , \end{aligned}$$

similarly, it can be continued to the following series

9 $$\begin{aligned} \vartheta (\Im ,\tau )=\vartheta _{0}+\vartheta _{1}+\vartheta _{2}+\cdots =\sum _{i=0}^{\infty }\vartheta _{i}(\Im ,\tau ). \end{aligned}$$

Equation (9) represents the approximate solution of the fractional problem (2).

## Convergence and error analysis

The following theorems are built on the idea of the proposed scheme and provided to show the convergence and error analysis of the problem (2)

### Theorem 4.1

*Let* $\vartheta (\Im , \tau )$ *be the exact results of Eq*. (2) *and consider* $\vartheta (\Im , \tau ), \vartheta _n(\Im , \tau ) \in H$ *and* $\sigma \in (0,1)$, *where* $\textrm{H}$ *represents the Hilbert space. Then, the derived results* $\sum _{i=0}^{\infty } \vartheta _i(\Im , \tau )$ *can converge* $\vartheta (\Im , \tau )$ *in case of* $\vartheta _i(\Im , \tau ) \le \vartheta _{i-1}(\Im , \tau ) \forall i>A$, *thus, for any* $\omega>0 \exists A>0$, *there is* $\left\| \vartheta _{i+n}(\Im , \tau )\right\| \le \beta , \forall m, n \in N$.

### *Proof*

Let a sequence such as $\sum _{i=0}^{\infty } \vartheta _i(\Im , \tau )$. Then

10 $$\begin{aligned} \begin{aligned} \vartheta _0(\Im , \tau )&=\vartheta _0(\Im , \tau ), \\ \vartheta _1(\Im , \tau )&=\vartheta _0(\Im , \tau )+\vartheta _1(\Im , \tau ), \\ \vartheta _2(\Im , {\mathfrak {I}})&=\vartheta _0(\Im , \tau )+\vartheta _1(\Im , \tau )+\vartheta _2(\Im , {\mathfrak {I}}), \\ \vartheta _3(\Im , {\mathfrak {I}})&=\vartheta _0(\Im , \tau )+\vartheta _1(\Im , \tau )+\vartheta _2(\Im , \tau )+\vartheta _3(\Im , \tau ), \\&\vdots \\ \vartheta _i(\Im , \tau )&=\vartheta _0(\Im , \tau )+\vartheta _1(\Im , \tau )+\vartheta _2(\Im , \tau )+\cdots +\vartheta _i(\Im , \tau ), \end{aligned} \end{aligned}$$

To achieve the valuable solution, we must show that $\vartheta _i(\Im , \tau )$ defines a “Cauchy sequence”. Moreover, consider

11 $$\begin{aligned} \begin{aligned} \left\| \vartheta _{i+1}(\Im , {\mathfrak {I}})-\vartheta _i(\Im , {\mathfrak {I}})\right\|&=\left\| \vartheta _{i+1}(\Im , {\mathfrak {I}})\right\| \le \sigma \left\| \vartheta _i(\Im , {\mathfrak {I}})\right\| \le \sigma ^2\left\| \vartheta _{i-1}(\Im , {\mathfrak {I}})\right\| \le \sigma ^3\left\| \vartheta _{i-2}(\Im , {\mathfrak {I}})\right\| \ldots \\&\le \sigma _{i+1}\left\| \vartheta _0(\Im , {\mathfrak {I}})\right\| . \end{aligned} \end{aligned}$$

For $i, n \in N$, it yields

12 $$\begin{aligned} \begin{aligned} \left\| \vartheta _i(\Im , \tau )-\vartheta _n(\Im , {\mathfrak {I}})\right\| =&\left\| \vartheta _{i+n}(\Im , \tau )\right\| =\Vert \vartheta _i(\Im , \tau )-\vartheta _{i-1}(\Im , \tau )+\left( \vartheta _{i-1}(\Im , \tau )-\vartheta _{i-2}(\Im , \tau )\right) \\&+\left( \vartheta _{i-2}(\Im , \tau )-\vartheta _{i-3}(\Im , \tau )\right) +\cdots +\left( \vartheta _{n+1}(\Im , \tau )-\vartheta _n(\Im , \tau )\right) \Vert , \\ \le&\left\| \vartheta _i(\Im , \tau )-\vartheta _{i-1}(\Im , \tau )\right\| +\left\| \left( \vartheta _{i-1}(\Im , {\mathfrak {I}})-\vartheta _{i-2}(\Im , {\mathfrak {I}})\right) \right\| \\&+\left\| \left( \vartheta _{i-2}(\Im , \tau )-\vartheta _{i-3}(\Im , \tau )\right) \right\| +\cdots +\Vert \left( \vartheta _{n+1}(\Im , \tau )-\vartheta _n(\Im , \tau ) \Vert \right. , \\ \le&\sigma ^i\left\| \vartheta _0(\Im , {\mathfrak {I}})\right\| +\sigma ^{i-1}\left\| \vartheta _0(\Im , \tau )\right\| +\cdots +\sigma ^{i+1}\left\| \vartheta _0(\Im , {\mathfrak {I}})\right\| , \\ =&\left\| \vartheta _0(\Im , \tau )\right\| \left( \sigma ^i+\sigma ^{i-1}+\sigma ^{i+1}\right) , \\ =&\left\| \vartheta _0(\Im , \tau )\right\| \frac{1-\sigma ^{i-n}}{1-\sigma ^{i+1}} \sigma ^{n+1}. \end{aligned} \end{aligned}$$

As $0<\sigma <1$, and $\vartheta _0(\Im , \tau )$ is bounded, then consider $\beta =1-\sigma /\left( 1-\sigma _{i-n}\right) \sigma ^{n+1}\left\| \vartheta _0(\Im , \tau )\right\|$, and thus, $\left\{ \vartheta _i(\Im , \tau )\right\} _{i=0}^{\infty }$ tends to “Cauchy sequence” in H. Hence, the sequence $\left\{ \vartheta _i(\Im , \tau )\right\} _{i=0}^{\infty }$ is convergent with the $\lim _{i\rightarrow \infty } \vartheta _i(\Im , \tau )=\vartheta (\Im , \tau )$ for $\exists \vartheta (\Im , \tau ) \in {\mathcal {H}}$. This ends the proof. $\square$

### Theorem 4.2

*Let* $\sum _{h=0}^k \vartheta _h(\Im , \tau )$ *is finite and* $\vartheta (\Im , \tau )$ *shows the derived series results. Consider* $\sigma >0$ *such as* $\left\| \vartheta _{h+1}(\Im , \tau )\right\| \le \left\| \vartheta _h(\Im , {\mathfrak {I}})\right\|$, *then the following relation produces the maximum absolute error*.

13 $$\begin{aligned} \left\| \vartheta (\Im , \tau )-\sum _{h=0}^k \vartheta _h(\Im , \tau )\right\| <\frac{\sigma ^{k+1}}{1-\sigma }\left\| \vartheta _0(\Im , \tau )\right\| . \end{aligned}$$

### *Proof*

Since $\sum _{h=0}^k \vartheta _h(\Im , \tau )$ is finite, this implies that $\sum _{h=0}^k \vartheta _h(\Im , \tau )<\infty$. Consider

14 $$\begin{aligned} \begin{aligned} \left\| \vartheta (\Im , \tau )-\sum _{h=0}^k \vartheta _h(\Im , {\mathfrak {I}})\right\|&=\left\| \sum _{h=k+1}^{\infty } \vartheta _h(\Im , \tau )\right\| , \\&\le \sum _{h=k+1}^{\infty }\left\| \vartheta _h(\Im , \tau )\right\| , \\&\le \sum _{h=k+1}^{\infty } \sigma ^h\left\| \vartheta _0(\Im , \tau )\right\| , \\&\le \sigma ^{k+1}\left( 1+\sigma +\sigma ^2+\cdots \right) \left\| \vartheta _0(\Im , \tau )\right\| , \\&\le \frac{\sigma ^{k+1}}{1-\sigma }\left\| \vartheta _0(\Im , \tau )\right\| . \end{aligned} \end{aligned}$$

This ends the proof. $\square$

## Applications

We illustrate four applications of multi-dimensional diffusion problems with time-fractional derivatives. We consider two-dimensional and three-dimensional heat flow problems in the sense of Caputo fractional derivative. These examples exhibit the performance and capability of the presented scheme. Graphical results and absolute errors show that YHPTM is a very promising tool for solving fractional differential problems. MATHEMATICA 11 software is used for numerical computations during the calculation phase and construction of figures.

### *Example 1*

Let us consider the two-dimensional homogeneous time-fractional heat flow problem

15 $$\begin{aligned} \frac{\partial ^{\alpha } \vartheta }{\partial \tau ^{\alpha }}=\frac{\partial ^{2}\vartheta }{\partial \Im ^{2}}+\frac{\partial ^{2}\vartheta }{\partial \wp ^{2}}-\vartheta , \end{aligned}$$

with the initial condition

16 $$\begin{aligned} \vartheta (\Im ,\wp ,0)=\sin \Im \cos \wp . \end{aligned}$$

Applying the YT on Eq. (15), we get

$$\begin{aligned} Y\Big [\frac{\partial ^{\alpha } \vartheta }{\partial \tau ^{\alpha }}\Big ]=Y\Big [\frac{\partial ^{2}\vartheta }{\partial \Im ^{2}}+\frac{\partial ^{2}\vartheta }{\partial \wp ^{2}}-\vartheta \Big ]. \end{aligned}$$

The application of YT in fractional form yields

$$\begin{aligned} \frac{1}{\xi ^{\alpha }}\Big [R(\xi )-\xi \vartheta (0)\Big ]=Y\Big [\frac{\partial ^{2}\vartheta }{\partial \Im ^{2}}+\frac{\partial ^{2}\vartheta }{\partial \wp ^{2}}-\vartheta \Big ]. \end{aligned}$$

Thus, $R(\xi )$ is obtained as

17 $$\begin{aligned} R[\xi ]=\xi \vartheta (0)+\xi ^{\alpha } Y\Big [\frac{\partial ^{2}\vartheta }{\partial \Im ^{2}}+\frac{\partial ^{2}\vartheta }{\partial \wp ^{2}}-\vartheta \Big ]. \end{aligned}$$

Using inverse YT on Eq. (17), we get

18 $$\begin{aligned} \vartheta (\Im ,\wp ,\tau )=\vartheta (\Im ,\wp ,0)+Y^{-1}\Big [\xi ^{\alpha } Y\Big [\frac{\partial ^{2}\vartheta }{\partial \Im ^{2}}+\frac{\partial ^{2}\vartheta }{\partial \wp ^{2}}-\vartheta \Big ]. \end{aligned}$$

Implementing the idea of of HPM to derive the He’s iterations

$$\begin{aligned} \sum _{i=0}^{\infty }p^{i}\vartheta (\Im ,\wp ,\tau )=\vartheta (\Im ,\wp ,0)+Y^{-1}\Big [\xi ^{\alpha } Y\Big [\sum _{i=0}^{\infty }p^{i}\frac{\partial ^{2}\vartheta _{i}}{\partial \Im ^{2}}+\sum _{i=0}^{\infty }p^{i}\frac{\partial ^{2}\vartheta _{i}}{\partial \wp ^{2}}-\sum _{i=0}^{\infty }p^{i}\vartheta _{i}\Big ]. \end{aligned}$$

Relating the similar components of *p*, we get

$$\begin{aligned} p^{0}&:\vartheta _{0}(\Im ,\wp ,\tau )=\vartheta (\Im ,\wp ,0)=\sin \Im \cos \wp ,\\ p^{1}&:\vartheta _{1}(\Im ,\wp ,\tau )=Y^{-1}\Bigg [\xi ^{\alpha } Y\bigg \{\frac{\partial ^{2}\vartheta _{0}}{\partial \Im ^{2}}+\frac{\partial ^{2}\vartheta _{0}}{\partial \wp ^{2}}-\vartheta _{0}\bigg \}\Bigg ]=-3 \sin \Im \cos \wp \frac{\tau ^{\alpha }}{\Gamma (\alpha +1)},\\ p^{2}&:\vartheta _{2}(\Im ,\wp ,\tau )=Y^{-1}\Bigg [\xi ^{\alpha } Y\bigg \{\frac{\partial ^{2}\vartheta _{1}}{\partial \Im ^{2}}+\frac{\partial ^{2}\vartheta _{1}}{\partial \wp ^{2}}-\vartheta _{1}\bigg \}\Bigg ]=3^{2} \sin \Im \cos \wp \frac{\tau ^{2\alpha }}{\Gamma (2\alpha +1)},\\ p^{3}&:\vartheta _{3}(\Im ,\wp ,\tau )=Y^{-1}\Bigg [\xi ^{\alpha } Y\bigg \{\frac{\partial ^{2}\vartheta _{2}}{\partial \Im ^{2}}+\frac{\partial ^{2}\vartheta _{2}}{\partial \wp ^{2}}-\vartheta _{2}\bigg \}\Bigg ]=-3^{3} \sin \Im \cos \wp \frac{\tau ^{3\alpha }}{\Gamma (3\alpha +1)},\\ p^{4}&:\vartheta _{4}(\Im ,\wp ,\tau )=Y^{-1}\Bigg [\xi ^{\alpha } Y\bigg \{\frac{\partial ^{2}\vartheta _{3}}{\partial \Im ^{2}}+\frac{\partial ^{2}\vartheta _{3}}{\partial \wp ^{2}}-\vartheta _{3}\bigg \}\Bigg ]=3^{4} \sin \Im \cos \wp \frac{\tau ^{4\alpha }}{\Gamma (4\alpha +1)},\\&\vdots . \end{aligned}$$

Similarly, it can be continued to the following series

19 $$\begin{aligned} \begin{aligned} \vartheta (\Im ,\wp ,\tau )&=\vartheta _{0}(\Im ,\wp ,\tau )+\vartheta _{1}(\Im ,\wp ,\tau )+\vartheta _{2}(\Im ,\wp ,\tau )+\vartheta _{3}(\Im ,\wp ,\tau )+\vartheta _{4}(\Im ,\wp ,\tau )+\cdots ,\\&=\sin \Im \cos \wp \Big (1-3\frac{\tau ^{\alpha }}{\Gamma (\alpha +1)}+3^{2}\frac{\tau ^{2\alpha }}{\Gamma (2\alpha +1)}-3^{3}\frac{\tau ^{3\alpha }}{\Gamma (3\alpha +1)}+3^{4}\frac{\tau ^{4\alpha }}{\Gamma (4\alpha +1)}+\cdots \Big )+\cdots . \end{aligned} \end{aligned}$$

which can be closed form

20 $$\begin{aligned} \vartheta (\Im ,\wp ,\tau )=e^{-3\tau }\sin \Im \cos \wp . \end{aligned}$$

**Figure 1:**
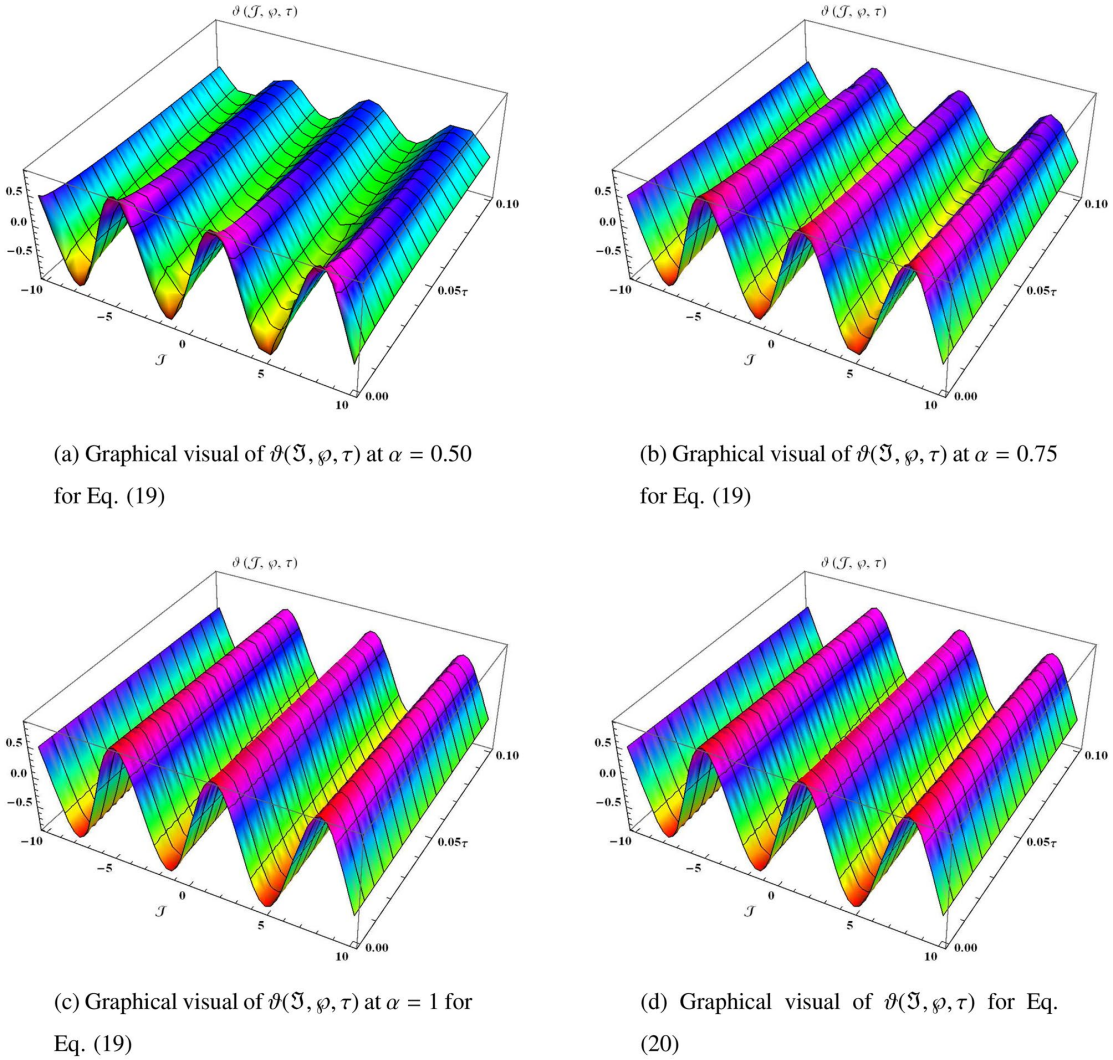
The three-dimensional surfaces solution of $\vartheta (\Im ,\wp ,\tau )$.

**Figure 2:**
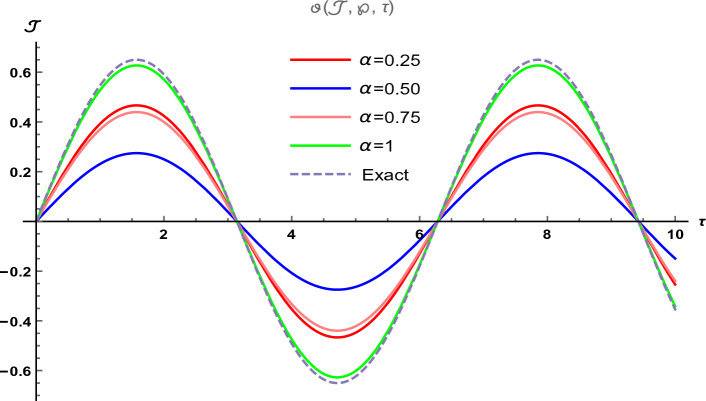
The two-dimensional graphical visual of $\vartheta (\Im ,\wp ,\tau )$ at multiple values of $\alpha$.

**Table 1:** Absolute error between the obtained results and the exact solution at $\wp =0.5$ and $\tau =0.001$.

$\Im$	$\alpha =0.50$	YHPTM results at $\alpha =1$	Exact results	Absolute error at $\alpha =0.50$	Absolute error at $\alpha =1$
0.25	0.194837	0.216466	0.216467	2.163$\times 10^{-2}$	1$\times 10^{-6}$
0.50	0.37756	0.419474	0.419475	4.1915$\times 10^{-2}$	1$\times 10^{-6}$
0.75	0.536807	0.59640	0.596402	5.9595$\times 10^{-2}$	1$\times 10^{-6}$
1.0	0.662679	0.736246	0.736248	7.3569$\times 10^{-2}$	1$\times 10^{-6}$
1.25	0.747349	0.830315	0.830318	8.2969$\times 10^{-2}$	1$\times 10^{-6}$
1.50	0.785552	0.872759	0.872762	8.7207$\times 10^{-2}$	1$\times 10^{-6}$
1.75	0.774913	0.860939	0.860942	8.6026$\times 10^{-2}$	1$\times 10^{-6}$
2.0	0.716094	0.795591	0.795593	7.9497$\times 10^{-2}$	1$\times 10^{-6}$

In Fig. 1, we provide the graphical visuals of approximate series solution of Eq. (19) and the exact solution of Eq. (20) at $-10 \le \Im \le 10$ and $0 \le \tau \le 0.1$. These visuals indicate that when we increase the value of fractional order $\alpha$, our graphical results approach to the exact graph significantly. We plotted the graphical error in two-dimensional visuals in Fig. 2 at $\alpha = 0.25, 0.50, 0.75, 1$. This shows comparison yields that YHPTM is fast and convenient approach. Table 1 presents the absolute errors between the approximate solution and the exact solution of three-dimensional heat flow problem. This table shows that when $\alpha =1$, our obtained values are very close to the exact solution than the values of $\alpha =0.50$ and the value of absolute error decreases precisely.

### *Example 2*

Consider the following time-fractional heat flow problem in a inhomogeneous two-dimensional form

21 $$\begin{aligned} \frac{\partial ^{\alpha } \vartheta }{\partial \tau ^{\alpha }}=\frac{\partial ^{2}\vartheta }{\partial \Im ^{2}}+\frac{\partial ^{2}\vartheta }{\partial \wp ^{2}}+\sin \wp , \end{aligned}$$

with the initial condition

22 $$\begin{aligned} \vartheta (\Im ,\wp ,0)=\sin \Im \sin \wp +\sin \wp . \end{aligned}$$

Applying the YT on Eq. (21), we get

$$\begin{aligned} Y\Big [\frac{\partial ^{\alpha } \vartheta }{\partial \tau ^{\alpha }}\Big ]=Y\Big [\frac{\partial ^{2}\vartheta }{\partial \Im ^{2}}+\frac{\partial ^{2}\vartheta }{\partial \wp ^{2}}+\sin \wp \Big ]. \end{aligned}$$

The application of YT in fractional form yields

$$\begin{aligned} \frac{1}{\xi ^{\alpha }}\Big [R(\xi )-\xi \vartheta (0)\Big ]=Y\Big [\frac{\partial ^{2}\vartheta }{\partial \Im ^{2}}+\frac{\partial ^{2}\vartheta }{\partial \wp ^{2}}+\sin \wp \Big ]. \end{aligned}$$

Thus $R(\xi )$ is obtained as

23 $$\begin{aligned} R[\xi ]=\xi \vartheta (0)+\xi ^{\alpha +1} \sin \wp +\xi ^{\alpha } Y\Big [\frac{\partial ^{2}\vartheta }{\partial \Im ^{2}}+\frac{\partial ^{2}\vartheta }{\partial \wp ^{2}}\Big ]. \end{aligned}$$

Using inverse YT on Eq. (23), we get

24 $$\begin{aligned} \vartheta (\Im ,\wp ,\tau )=\vartheta (\Im ,\wp ,0)+\sin \wp \frac{\tau ^{\alpha }}{\Gamma (\alpha +1)} +Y^{-1}\Big [\xi ^{\alpha } Y\Big [\frac{\partial ^{2}\vartheta }{\partial \Im ^{2}}+\frac{\partial ^{2}\vartheta }{\partial \wp ^{2}}\Big ]. \end{aligned}$$

Implementing the idea of HPM to derive the He’s iterations

$$\begin{aligned} \sum _{i=0}^{\infty }p^{i}\vartheta (\Im ,\wp ,\tau )=\vartheta (\Im ,\wp ,0)+\sin \wp \frac{\tau ^{\alpha }}{\Gamma (\alpha +1)}+Y^{-1}\Big [\xi ^{\alpha } Y\Big [\sum _{i=0}^{\infty }p^{i}\frac{\partial ^{2}\vartheta _{i}}{\partial \Im ^{2}}+\sum _{i=0}^{\infty }p^{i}\frac{\partial ^{2}\vartheta _{i}}{\partial \wp ^{2}}\Big ]. \end{aligned}$$

Relating the similar components of *p*, we get

$$\begin{aligned} p^{0}&:\vartheta _{0}(\Im ,\wp ,\tau )=\vartheta (\Im ,\wp ,0)=\sin \Im \sin \wp +\sin \wp +\sin \wp \frac{\tau ^{\alpha }}{\Gamma (\alpha +1)},\\ p^{1}&:\vartheta _{1}(\Im ,\wp ,\tau )=Y^{-1}\Bigg [\xi ^{\alpha } Y\bigg \{\frac{\partial ^{2}\vartheta _{0}}{\partial \Im ^{2}}+\frac{\partial ^{2}\vartheta _{0}}{\partial \wp ^{2}}\bigg \}\Bigg ]=-2\sin \Im \sin \wp \frac{\tau ^{\alpha }}{\Gamma (\alpha +1)}-\sin \wp \frac{\tau ^{\alpha }}{\Gamma (\alpha +1)}-\sin \wp \frac{\tau ^{2\alpha }}{\Gamma (2\alpha +1)},\\ p^{2}&:\vartheta _{2}(\Im ,\wp ,\tau )=Y^{-1}\Bigg [\xi ^{\alpha } Y\bigg \{\frac{\partial ^{2}\vartheta _{1}}{\partial \Im ^{2}}+\frac{\partial ^{2}\vartheta _{1}}{\partial \wp ^{2}}\bigg \}\Bigg ]=2^{2}\sin \Im \sin \wp \frac{\tau ^{2\alpha }}{\Gamma (2\alpha +1)}+\sin \wp \frac{\tau ^{2\alpha }}{\Gamma (2\alpha +1)}+\sin \wp \frac{\tau ^{3\alpha }}{\Gamma (3\alpha +1)},\\ p^{3}&:\vartheta _{3}(\Im ,\wp ,\tau )=Y^{-1}\Bigg [\xi ^{\alpha } Y\bigg \{\frac{\partial ^{2}\vartheta _{2}}{\partial \Im ^{2}}+\frac{\partial ^{2}\vartheta _{2}}{\partial \wp ^{2}}\bigg \}\Bigg ]=-2^{3}\sin \Im \sin \wp \frac{\tau ^{3\alpha }}{\Gamma (3\alpha +1)}-\sin \wp \frac{\tau ^{3\alpha }}{\Gamma (3\alpha +1)}-\sin \wp \frac{\tau ^{4\alpha }}{\Gamma (4\alpha +1)},\\ p^{4}&:\vartheta _{4}(\Im ,\wp ,\tau )=Y^{-1}\Bigg [\xi ^{\alpha } Y\bigg \{\frac{\partial ^{2}\vartheta _{3}}{\partial \Im ^{2}}+\frac{\partial ^{2}\vartheta _{3}}{\partial \wp ^{2}}\bigg \}\Bigg ]=-2^{4}\sin \Im \sin \wp \frac{\tau ^{4\alpha }}{\Gamma (4\alpha +1)}-\sin \wp \frac{\tau ^{4\alpha }}{\Gamma (4\alpha +1)}-\sin \wp \frac{\tau ^{5\alpha }}{\Gamma (5\alpha +1)},\\&\vdots . \end{aligned}$$

Similarly, it can be continued to the following series

25 $$\begin{aligned} \begin{aligned} \vartheta (\Im ,\wp ,\tau )&=\vartheta _{0}(\Im ,\wp ,\tau )+\vartheta _{1}(\Im ,\wp ,\tau )+\vartheta _{2}(\Im ,\wp ,\tau )+\vartheta _{3}(\Im ,\wp ,\tau )+\vartheta _{4}(\Im ,\wp ,\tau )+\cdots ,\\&=\sin \wp +\sin \Im \sin \wp \Big (1-2\frac{\tau ^{\alpha }}{\Gamma (\alpha +1)}+2^{2}\frac{\tau ^{2\alpha }}{\Gamma (2\alpha +1)}-2^{3}\frac{\tau ^{3\alpha }}{\Gamma (3\alpha +1)}+2^{4}\frac{\tau ^{4\alpha }}{\Gamma (4\alpha +1)}+\cdots \Big )+{\cdots ,} \end{aligned} \end{aligned}$$

which can be closed form

26 $$\begin{aligned} \vartheta (\Im ,\wp ,\tau )=\sin \wp +e^{-2\tau }\sin \Im \sin \wp . \end{aligned}$$

**Figure 3:**
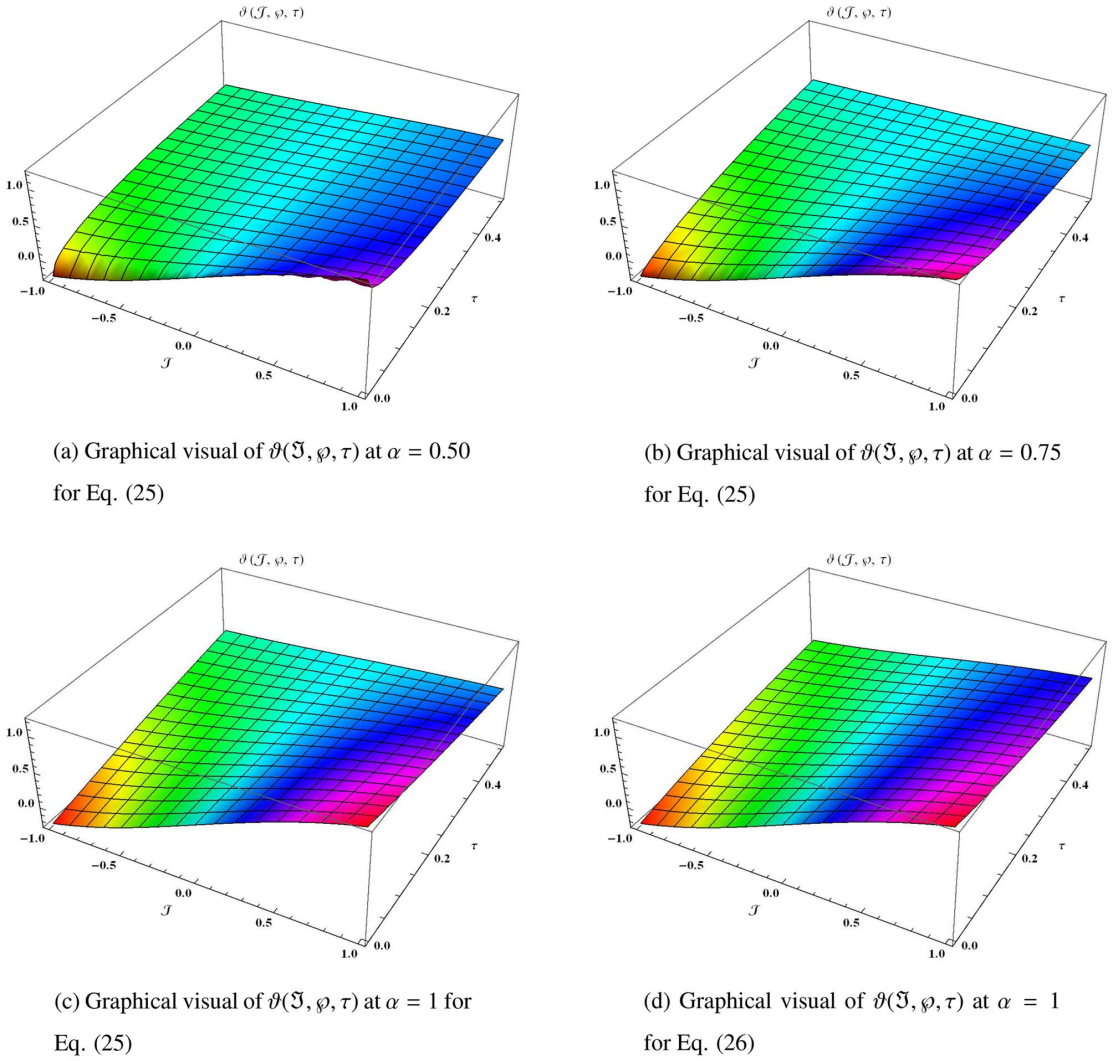
The three-dimensional surfaces solution of $\vartheta (\Im ,\wp ,\tau )$.

**Figure 4:**
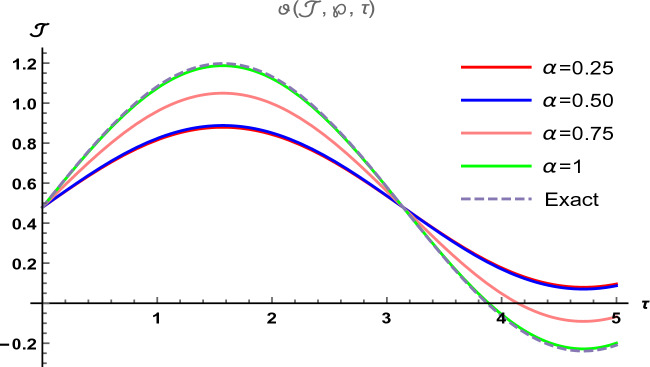
The two-dimensional graphical visual of $\vartheta (\Im ,\wp ,\tau )$ at multiple values of $\alpha$.

**Table 2:** Absolute error between the obtained results and the exact solution at $\wp =0.5$ and $\tau =0.005$.

$\Im$	$\alpha =0.50$	YHPTM results at $\alpha =1$	Exact results	Absolute error at $\alpha =0.50$	Absolute error at $\alpha =1$
0.25	0.681478	0.696109	0.696109	1.4631$\times 10^{-2}$	0.00000
0.50	0.870968	0.89932	0.89932	2.8352$\times 10^{-2}$	0.00000
0.75	1.03611	1.07642	1.07642	4.031$\times 10^{-2}$	0.00000
1.0	1.16665	1.21641	1.21641	4.976$\times 10^{-2}$	0.00000
1.25	1.25445	1.31057	1.31057	5.612$\times 10^{-2}$	0.00000
1.50	1.29407	1.35306	1.35306	5.899$\times 10^{-2}$	0.00000
1.75	1.28304	1.34123	1.34123	5.819$\times 10^{-2}$	0.00000
2.0	1.22204	1.27581	1.27581	5.377$\times 10^{-2}$	0.00000

In Fig. 3, we provide the graphical visuals of approximate series solution of Eq. (25) and the exact solution of Eq. (26) at $-1 \le \Im \le 1$ and $0 \le \tau \le 0.5$. These visuals indicate that when we increase the value of fractional order $\alpha$, our graphical results approach to the exact graph significantly. We plotted the graphical error in two-dimensional visuals in Fig. 4 at $\alpha = 0.25, 0.50, 0.75, 1$. This shows comparison yields that YHPTM is fast and convenient approach. Table 2 presents the absolute errors between the approximate solution and the exact solution of three-dimensional heat flow problem. This table shows that when $\alpha =1$, our obtained values are very close to the exact solution than the values of $\alpha =0.50$ and the value of absolute error decreases precisely.

### Example 3

Consider the following time-fractional heat flow problem in a three-dimensional homogeneous form

27 $$\begin{aligned} \frac{\partial ^{\alpha } \vartheta }{\partial \tau ^{\alpha }}=\frac{\partial ^{2}\vartheta }{\partial \Im ^{2}}+\frac{\partial ^{2}\vartheta }{\partial \wp ^{2}}+\frac{\partial ^{2}\vartheta }{\partial \varpi ^{2}}-2\vartheta , \end{aligned}$$

with the initial condition

28 $$\begin{aligned} \vartheta (\Im ,\wp ,\varpi ,0)=\sin \Im \sin \wp \sin \varpi . \end{aligned}$$

Applying the YT on Eq. (27), we get

$$\begin{aligned} Y\Big [\frac{\partial ^{\alpha } \vartheta }{\partial \tau ^{\alpha }}\Big ]=Y\Big [\frac{\partial ^{2}\vartheta }{\partial \Im ^{2}}+\frac{\partial ^{2}\vartheta }{\partial \wp ^{2}}+\frac{\partial ^{2}\vartheta }{\partial \varpi ^{2}}-2\vartheta \Big ]. \end{aligned}$$

Using the properties functions of YT , we obtain

$$\begin{aligned} \frac{1}{\xi ^{\alpha }}\Big [R(\xi )-\xi \vartheta (0)\Big ]=Y\Big [\frac{\partial ^{2}\vartheta }{\partial \Im ^{2}}+\frac{\partial ^{2}\vartheta }{\partial \wp ^{2}}+\frac{\partial ^{2}\vartheta }{\partial \varpi ^{2}}-2\vartheta \Big ]. \end{aligned}$$

Thus $R(\xi )$ is obtained as

29 $$\begin{aligned} R[\xi ]=\xi \vartheta (0)+\xi ^{\alpha } Y\Big [\frac{\partial ^{2}\vartheta }{\partial \Im ^{2}}+\frac{\partial ^{2}\vartheta }{\partial \wp ^{2}}+\frac{\partial ^{2}\vartheta }{\partial \varpi ^{2}}-2\vartheta \Big ]. \end{aligned}$$

Using inverse YT on Eq. (29), we get

30 $$\begin{aligned} \vartheta (\Im ,\wp ,\varpi ,\tau )=\vartheta (\Im ,\wp ,0)+Y^{-1}\Big [\xi ^{\alpha } Y\Big [\frac{\partial ^{2}\vartheta }{\partial \Im ^{2}}+\frac{\partial ^{2}\vartheta }{\partial \wp ^{2}}+\frac{\partial ^{2}\vartheta }{\partial \varpi ^{2}}-2\vartheta \Big ]. \end{aligned}$$

Implementing the idea of HPM to derive the He’s iterations

$$\begin{aligned} \sum _{i=0}^{\infty }p^{i}\vartheta _{i}(\Im ,\wp ,\varpi ,\tau )=\vartheta (\Im ,\wp ,\varpi ,0)+Y^{-1}\Big [\xi ^{\alpha } Y\Big [\sum _{i=0}^{\infty }p^{i}\frac{\partial ^{2}\vartheta _{i}}{\partial \Im ^{2}}+\sum _{i=0}^{\infty }p^{i}\frac{\partial ^{2}\vartheta _{i}}{\partial \wp ^{2}}+\sum _{i=0}^{\infty }p^{i}\frac{\partial ^{2}\vartheta _{i}}{\partial \varpi ^{2}}-2\sum _{i=0}^{\infty }p^{i}\vartheta _{i}\Big ]. \end{aligned}$$

Relating the similar components of *p*, we get

$$\begin{aligned} p^{0}&:\vartheta _{0}(\Im ,\wp ,\varpi ,\tau )=\vartheta (\Im ,\wp ,\varpi ,0)=\sin \Im \sin \wp \sin \varpi ,\\ p^{1}&:\vartheta _{1}(\Im ,\wp ,\varpi ,\tau )=Y^{-1}\Bigg [\xi ^{\alpha } Y\bigg \{\frac{\partial ^{2}\vartheta _{0}}{\partial \Im ^{2}}+\frac{\partial ^{2}\vartheta _{0}}{\partial \wp ^{2}}+\frac{\partial ^{2}\vartheta _{3}}{\partial \varpi ^{2}}-2\vartheta _{0}\bigg \}\Bigg ]=-5\sin \Im \sin \wp \sin \varpi \frac{\tau ^{\alpha }}{\Gamma (\alpha +1)},\\ p^{2}&:\vartheta _{2}(\Im ,\wp ,\varpi ,\tau )=Y^{-1}\Bigg [\xi ^{\alpha } Y\bigg \{\frac{\partial ^{2}\vartheta _{1}}{\partial \Im ^{2}}+\frac{\partial ^{2}\vartheta _{1}}{\partial \wp ^{2}}+\frac{\partial ^{2}\vartheta _{3}}{\partial \varpi ^{2}}-2\vartheta _{1}\bigg \}\Bigg ]=5^{2}\sin \Im \sin \wp \sin \varpi \frac{\tau ^{2\alpha }}{\Gamma (2\alpha +1)},\\ p^{3}&:\vartheta _{3}(\Im ,\wp ,\varpi ,\tau )=Y^{-1}\Bigg [\xi ^{\alpha } Y\bigg \{\frac{\partial ^{2}\vartheta _{2}}{\partial \Im ^{2}}+\frac{\partial ^{2}\vartheta _{2}}{\partial \wp ^{2}}+\frac{\partial ^{2}\vartheta _{3}}{\partial \varpi ^{2}}-2\vartheta _{2}\bigg \}\Bigg ]=-5^{3}\sin \Im \sin \wp \sin \varpi \frac{\tau ^{3\alpha }}{\Gamma (3\alpha +1)},\\ p^{4}&:\vartheta _{4}(\Im ,\wp ,\varpi ,\tau )=Y^{-1}\Bigg [\xi ^{\alpha } Y\bigg \{\frac{\partial ^{2}\vartheta _{3}}{\partial \Im ^{2}}+\frac{\partial ^{2}\vartheta _{3}}{\partial \wp ^{2}}+\frac{\partial ^{2}\vartheta _{3}}{\partial \varpi ^{2}}-2\vartheta _{3}\bigg \}\Bigg ]=5^{4}\sin \Im \sin \wp \sin \varpi \frac{\tau ^{4\alpha }}{\Gamma (4\alpha +1)},\\&\vdots . \end{aligned}$$

Similarly, it can be continued to the following series

31 $$\begin{aligned} \begin{aligned} \vartheta (\Im ,\wp ,\varpi ,\tau )&=\vartheta _{0}(\Im ,\wp ,\varpi ,\tau )+\vartheta _{1}(\Im ,\wp ,\varpi ,\tau )+\vartheta _{2}(\Im ,\wp ,\varpi ,\tau )+\vartheta _{3}(\Im ,\wp ,\varpi ,\tau )+\vartheta _{4}(\Im ,\wp ,\varpi ,\tau )+\cdots ,\\&=\sin \Im \sin \wp \sin \varpi \Big (1-5\frac{\tau ^{\alpha }}{\Gamma (\alpha +1)}+5^{2}\frac{\tau ^{2\alpha }}{\Gamma (2\alpha +1)}-5^{3}\frac{\tau ^{3\alpha }}{\Gamma (3\alpha +1)}+5^{4}\frac{\tau ^{4\alpha }}{\Gamma (4\alpha +1)}+\cdots \Big )+\cdots {,} \end{aligned} \end{aligned}$$

which can be closed form

32 $$\begin{aligned} \vartheta (\Im ,\wp ,\varpi ,\tau )=e^{-5\tau }\sin \Im \sin \wp \sin \varpi . \end{aligned}$$

**Figure 5:**
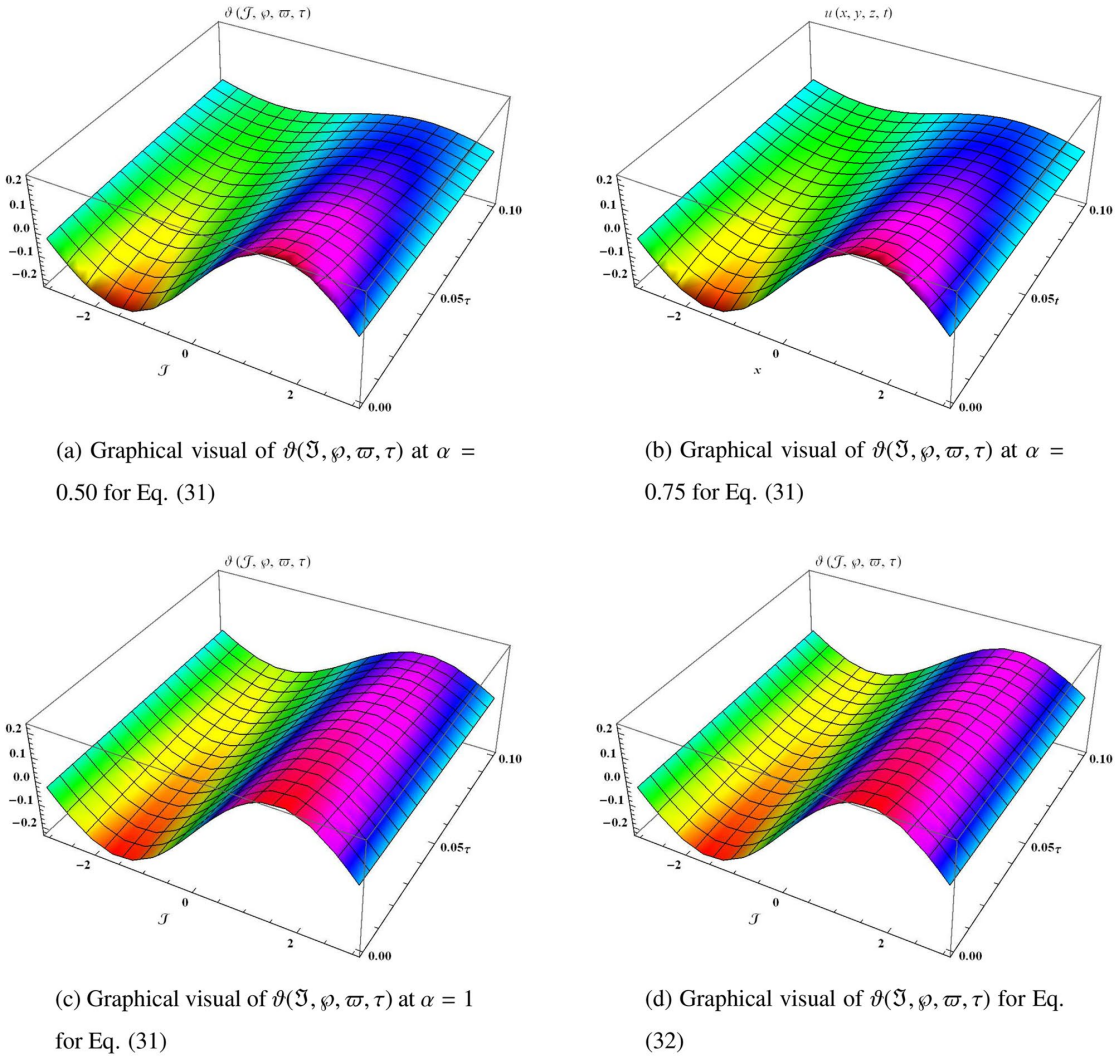
The three-dimensional surfaces solution of $\vartheta (\Im ,\wp ,\varpi ,\tau )$.

**Figure 6:**
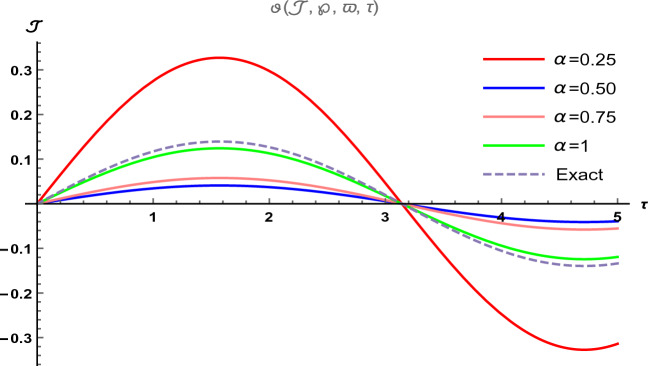
The two-dimensional graphical visual of $\vartheta (\Im ,\wp ,\varpi ,\tau )$ at multiple values of $\alpha$.

**Table 3:** Absolute error between the obtained results and the exact solution at $\wp =\varpi =0.5$ and $\tau =0.001$.

$\Im$	$\alpha =0.50$	YHPTM results at $\alpha =1$	Exact results	Absolute error at $\alpha =0.50$	Absolute error at $\alpha =1$
0.25	0.0375249	0.0554498	0.0554615	1.79366$\times 10^{-2}$	1.17$\times 10^{-5}$
0.50	0.0727167	0.107452	0.107475	3.47581$\times 10^{-2}$	2.3$\times 10^{-5}$
0.75	0.103387	0.152773	0.152806	4.9419$\times 10^{-2}$	3.3$\times 10^{-5}$
1.0	0.12763	0.188596	0.188636	6.1006$\times 10^{-2}$	4.0$\times 10^{-5}$
1.25	0.143937	0.212693	0.212738	6.8801$\times 10^{-2}$	4.5$\times 10^{-5}$
1.50	0.151295	0.223565	0.223612	7.2317$\times 10^{-2}$	4.7$\times 10^{-5}$
1.75	0.149246	0.220537	0.220584	7.1338$\times 10^{-2}$	4.7$\times 10^{-5}$
2.0	0.137917	0.203798	0.203841	6.5924$\times 10^{-2}$	4.3$\times 10^{-5}$

In Fig. 5, we provide the graphical visuals of approximate series solution of Eq. (31) and the exact solution of Eq. (32) $-3 \le \Im \le 3$ and $0 \le \tau \le 0.1$. These visuals indicate that when we increase the value of fractional order $\alpha$, our graphical results approach to the exact graph significantly. We plotted the graphical error in two-dimensional visuals in Fig. 6 at $\alpha = 0.25, 0.50, 0.75, 1$. This shows comparison yields that YHPTM is fast and convenient approach. Table 3 presents the absolute errors between the approximate solution and the exact solution of three-dimensional heat flow problem. This table shows that when $\alpha =1$, our obtained values are very close to the exact solution than the values of $\alpha =0.50$ and the value of absolute error decreases precisely.

### *Example 4*

Consider the following time-fractional heat flow problem in a three-dimensional inhomogeneous form

33 $$\begin{aligned} \frac{\partial ^{\alpha } \vartheta }{\partial \tau ^{\alpha }}=\frac{\partial ^{2}\vartheta }{\partial \Im ^{2}}+\frac{\partial ^{2}\vartheta }{\partial \wp ^{2}}+\frac{\partial ^{2}\vartheta }{\partial \varpi ^{2}}+\sin \varpi , \end{aligned}$$

with the initial condition

34 $$\begin{aligned} \vartheta (\Im ,\wp ,\varpi ,0)=\sin (\Im +\wp )+\sin \varpi . \end{aligned}$$

The application of YT in fractional form yields

$$\begin{aligned} Y\Big [\frac{\partial ^{\alpha } \vartheta }{\partial \tau ^{\alpha }}\Big ]=Y\Big [\frac{\partial ^{2}\vartheta }{\partial \Im ^{2}}+\frac{\partial ^{2}\vartheta }{\partial \wp ^{2}}+\frac{\partial ^{2}\vartheta }{\partial \varpi ^{2}}+\sin \varpi \Big ]. \end{aligned}$$

Using the properties functions of YT, we obtain

$$\begin{aligned} \frac{1}{\xi ^{\alpha }}\Big [R(\xi )-\xi \vartheta (0)\Big ]=Y\Big [\frac{\partial ^{2}\vartheta }{\partial \Im ^{2}}+\frac{\partial ^{2}\vartheta }{\partial \wp ^{2}}+\frac{\partial ^{2}\vartheta }{\partial \varpi ^{2}}+\sin \varpi \Big ]. \end{aligned}$$

Thus $R(\xi )$ is obtained as

35 $$\begin{aligned} R[\xi ]=\xi \vartheta (0)+\xi ^{\alpha } \wp [\sin \varpi ]+\xi ^{\alpha } Y\Big [\frac{\partial ^{2}\vartheta }{\partial \Im ^{2}}+\frac{\partial ^{2}\vartheta }{\partial \wp ^{2}}+\frac{\partial ^{2}\vartheta }{\partial \varpi ^{2}}\Big ]. \end{aligned}$$

Using inverse YT on Eq. (35), we get

36 $$\begin{aligned} \vartheta (\Im ,\wp ,\varpi ,\tau )=\vartheta (\Im ,\wp ,\varpi ,0)+\sin \varpi \frac{\tau ^{\alpha }}{\Gamma (\alpha +1)}+Y^{-1}\Big [\xi ^{\alpha } Y\Big [\frac{\partial ^{2}\vartheta }{\partial \Im ^{2}}+\frac{\partial ^{2}\vartheta }{\partial \wp ^{2}}+\frac{\partial ^{2}\vartheta }{\partial \varpi ^{2}}\Big ]. \end{aligned}$$

Implementing the idea of HPM to derive the He’s iterations

$$\begin{aligned} \sum _{i=0}^{\infty }p^{i}\vartheta (\Im ,\wp ,\varpi ,\tau )=\vartheta (\Im ,\wp ,\varpi ,0)+\sin \varpi \frac{\tau ^{\alpha }}{\Gamma (\alpha +1)}+Y^{-1}\Big [\xi ^{\alpha } Y\Big [\sum _{i=0}^{\infty }p^{i}\frac{\partial ^{2}\vartheta _{i}}{\partial \Im ^{2}}+\sum _{i=0}^{\infty }p^{i}\frac{\partial ^{2}\vartheta _{i}}{\partial \wp ^{2}}+\sum _{i=0}^{\infty }p^{i}\frac{\partial ^{2}\vartheta _{i}}{\partial \varpi ^{2}}\Big ]. \end{aligned}$$

Relating the similar components of *p*, we get

$$\begin{aligned} p^{0}&:\vartheta _{0}(\Im ,\wp ,\varpi ,\tau )=\vartheta (\Im ,\wp ,0)=\sin (\Im +\wp )+\sin \varpi +\sin \varpi \frac{\tau ^{\alpha }}{\Gamma (\alpha +1)},\\ p^{1}&:\vartheta _{1}(\Im ,\wp ,\varpi ,\tau )=Y^{-1}\Bigg [\xi ^{\alpha } Y\bigg \{\frac{\partial ^{2}\vartheta _{0}}{\partial \Im ^{2}}+\frac{\partial ^{2}\vartheta _{0}}{\partial \wp ^{2}}+\frac{\partial ^{2}\vartheta _{0}}{\partial \varpi ^{2}}\bigg \}\Bigg ]=-2 \sin (\Im +\wp )\frac{\tau ^{\alpha }}{\Gamma (\alpha +1)}-\sin \varpi \frac{\tau ^{\alpha }}{\Gamma (\alpha +1)}-\sin \varpi \frac{\tau ^{2\alpha }}{\Gamma (2\alpha +1)},\\ p^{2}&:\vartheta _{2}(\Im ,\wp ,\varpi ,\tau )=Y^{-1}\Bigg [\xi ^{\alpha } Y\bigg \{\frac{\partial ^{2}\vartheta _{1}}{\partial \Im ^{2}}+\frac{\partial ^{2}\vartheta _{1}}{\partial \wp ^{2}}+\frac{\partial ^{2}\vartheta _{0}}{\partial \varpi ^{2}}\bigg \}\Bigg ]=2^{2} \sin (\Im +\wp )\frac{\tau ^{2\alpha }}{\Gamma (2\alpha +1)}+\sin \varpi \frac{\tau ^{2\alpha }}{\Gamma (2\alpha +1)}+\sin \varpi \frac{\tau ^{3\alpha }}{\Gamma (3\alpha +1)},\\ p^{3}&:\vartheta _{3}(\Im ,\wp ,\varpi ,\tau )=Y^{-1}\Bigg [\xi ^{\alpha } Y\bigg \{\frac{\partial ^{2}\vartheta _{2}}{\partial \Im ^{2}}+\frac{\partial ^{2}\vartheta _{2}}{\partial \wp ^{2}}\bigg \}+\frac{\partial ^{2}\vartheta _{0}}{\partial \varpi ^{2}}\Bigg ]=-2^{3} \sin (\Im +\wp )\frac{\tau ^{3\alpha }}{\Gamma (3\alpha +1)}-\sin \varpi \frac{\tau ^{3\alpha }}{\Gamma (3\alpha +1)}-\sin \varpi \frac{\tau ^{4\alpha }}{\Gamma (4\alpha +1)},\\ p^{4}&:\vartheta _{4}(\Im ,\wp ,\varpi ,\tau )=Y^{-1}\Bigg [\xi ^{\alpha } Y\bigg \{\frac{\partial ^{2}\vartheta _{3}}{\partial \Im ^{2}}+\frac{\partial ^{2}\vartheta _{3}}{\partial \wp ^{2}}+\frac{\partial ^{2}\vartheta _{0}}{\partial \varpi ^{2}}\bigg \}\Bigg ]=2^{4} \sin (\Im +\wp )\frac{\tau ^{4\alpha }}{\Gamma (4\alpha +1)}+\sin \varpi \frac{\tau ^{4\alpha }}{\Gamma (4\alpha +1)}+\sin \varpi \frac{\tau ^{5\alpha }}{\Gamma (5\alpha +1)},\\&\vdots . \end{aligned}$$

Similarly, it can be continued to the following series

37 $$\begin{aligned} \begin{aligned} \vartheta (\Im ,\wp ,\varpi ,\tau )&=\vartheta _{0}(\Im ,\wp ,\varpi ,\tau )+\vartheta _{1}(\Im ,\wp ,\varpi ,\tau )+\vartheta _{2}(\Im ,\wp ,\varpi ,\tau )+\vartheta _{3}(\Im ,\wp ,\varpi ,\tau )+\vartheta _{4}(\Im ,\wp ,\varpi ,\tau )+\cdots ,\\&=\sin \varpi +\sin (\Im +\wp ) \left( 1-2\frac{\tau ^{\alpha }}{\Gamma (\alpha +1)}+2^{2}\frac{\tau ^{2\alpha }}{\Gamma (2\alpha +1)}-2^{3}\frac{\tau ^{3\alpha }}{\Gamma (3\alpha +1)}+2^{4}\frac{\tau ^{4\alpha }}{\Gamma (4\alpha +1)}+\cdots \right) + {\cdots ,} \end{aligned} \end{aligned}$$

which can be closed form

38 $$\begin{aligned} \vartheta (\Im ,\wp ,\varpi ,\tau )=\sin \varpi +e^{-2\tau }\sin (\Im +\wp ). \end{aligned}$$

**Figure 7:**
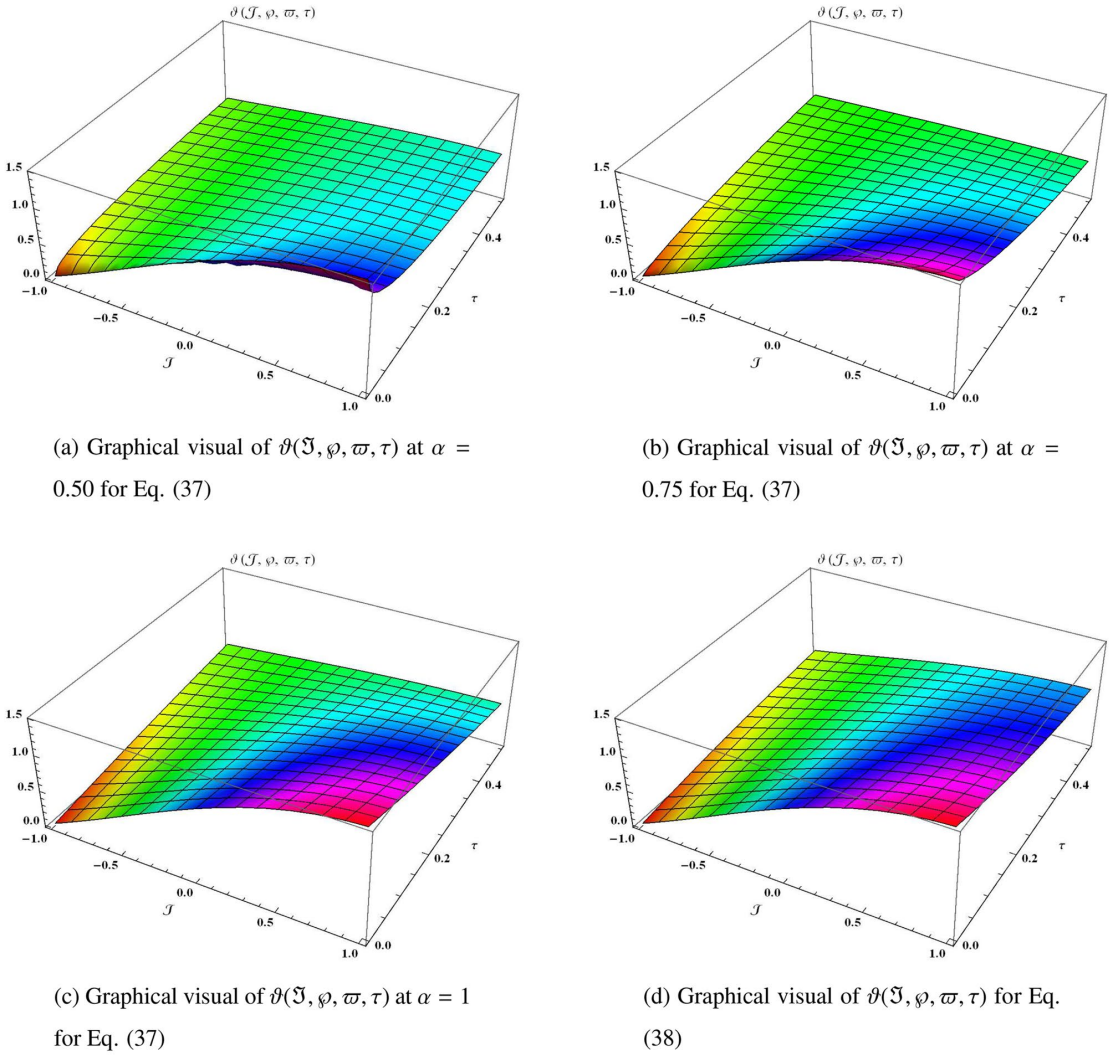
The three-dimensional surfaces solution of $\vartheta (\Im ,\wp ,\varpi ,\tau )$.

**Figure 8:**
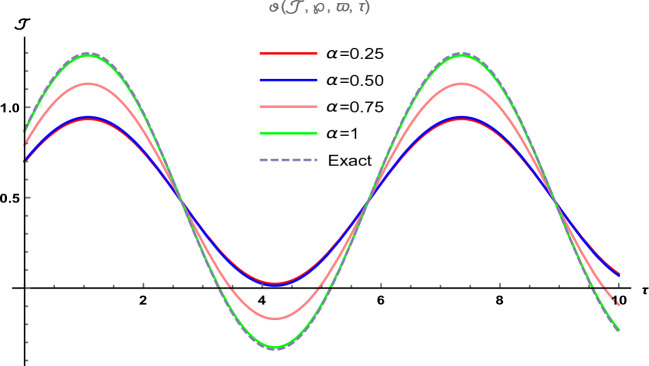
The two-dimensional graphical visual of $\vartheta (\Im ,\wp ,\varpi ,\tau )$ at multiple values of $\alpha$.

**Table 4:** Absolute error between the obtained results and the exact solution at $\wp =\varpi =0.5$ and $\tau =0.005$.

$\Im$	$\alpha =0.50$	YHPTM results at $\alpha =1$	Exact results	Absolute error at $\alpha =0.50$	Absolute error at $\alpha =1$
0.25	1.05894	1.15426	1.15428	9.534$\times 10^{-5}$	2$\times 10^{-2}$
0.50	1.19483	1.31250	1.31252	1.1769$\times 10^{-2}$	2$\times 10^{-5}$
0.75	1.28624	1.41894	1.41897	1.3273$\times 10^{-2}$	3$\times 10^{-5}$
1.0	1.32748	1.46696	1.4670	1.3952$\times 10^{-2}$	4$\times 10^{-5}$
1.25	1.31599	1.45359	1.45362	1.3763$\times 10^{-2}$	3$\times 10^{-5}$
1.50	1.25249	1.37965	1.37968	1.2719$\times 10^{-2}$	3$\times 10^{-5}$
1.75	1.14093	1.24973	1.24976	1.0883$\times 10^{-2}$	3$\times 10^{-5}$
2.0	0.988237	1.07192	1.07194	8.3703$\times 10^{-2}$	2$\times 10^{-5}$

In Fig. 7, we provide the graphical visuals of approximate series solution of Eq. (37) and the exact solution of Eq. (38) $-1 \le \Im \le 1$ and $0 \le \tau \le 0.5$. These visuals indicate that when we increase the value of fractional order $\alpha$, our graphical results approach to the exact graph significantly. We plotted the graphical error in two-dimensional visuals in Fig. 8 at $\alpha = 0.25, 0.50, 0.75, 1$. This shows comparison yields that YHPTM is fast and convenient approach. Table 4 presents the absolute errors between the approximate solution and the exact solution of three-dimensional heat flow problem. This table shows that when $\alpha =1$, our obtained values are very close to the exact solution than the values of $\alpha =0.50$ and the value of absolute error decreases precisely.

## Conclusion

In this study, we successfully developed the YHPTM approach for obtaining the approximate solution of the two-dimensional and three-dimensional heat flow problems. Since the equations involving fractional order are quite difficult to solve directly, we introduce the idea of YT to dissolve the fractional order of the problem. The scheme of YT is limited and unable to generate the series solution, therefore, we implement HPM to derive the successive iterations from the classical equation that leads the results to the exact solution very easily. We consider four test problems to show the efficiency and effectiveness of this proposed scheme. It has been found that our derived results demonstrate a great confirmation of compromise with the exact solution. We also analyzed the efficiency of our proposed scheme in two-dimensional and three-dimensional through graphical structures. The obtained results are efficient and significant, demonstrating that YHPTM is accurate and authentic for fractional problems. It is expected to consider this scheme for fractional problems in the sense of Atangana–Baleanu derivatives and other partial differential equations involving fractal theory and fractional calculus in our future work.

## Data Availability

This article contains all the data within the study.

## References

[1] Bayrak MA, Demir A (2018). A new approach for space-time fractional partial differential equations by residual power series method. Appl. Math. Comput..

[2] Alaoui MK, Fayyaz R, Khan A, Shah R, Abdo MS (2021). Analytical investigation of noyes-field model for time-fractional Belousov–Zhabotinsky reaction. Complexity.

[3] Malan A, Lewis R (2011). An artificial compressibility cbs method for modelling heat transfer and fluid flow in heterogeneous porous materials. Int. J. Numer. Methods Eng..

[4] Arafa AA, Hagag AMS (2019). A new analytic solution of fractional coupled Ramani equation. Chin. J. Phys..

[5] El-Sayed A, Rida S, Arafa A (2010). On the solutions of the generalized reaction–diffusion model for bacterial colony. Acta Appl. Math..

[6] Arafa AA, El-Sayed AM, Hagag AMSH (2021). A fractional Temimi–Ansari method (ftam) with convergence analysis for solving physical equations. Math. Methods Appl. Sci..

[7] Arikoglu A, Ozkol I (2007). Solution of fractional differential equations by using differential transform method. Chaos Solitons Fractals.

[8] Li Y, Zhao W (2010). Haar wavelet operational matrix of fractional order integration and its applications in solving the fractional order differential equations. Appl. Math. Comput..

[9] Rida S, Arafa A, Abedl-Rady A, Abdl-Rahaim H (2017). Fractional physical differential equations via natural transform. Chin. J. Phys..

[10] Dubey VP, Singh J, Alshehri AM, Dubey S, Kumar D (2022). Forecasting the behavior of fractional order Bloch equations appearing in nmr flow via a hybrid computational technique. Chaos Solitons Fractals.

[11] Li C, Zeng F (2013). The finite difference methods for fractional ordinary differential equations. Numer. Funct. Anal. Optim..

[12] Jiang Y, Ma J (2011). High-order finite element methods for time-fractional partial differential equations. J. Comput. Appl. Math..

[13] Dubey S, Dubey VP, Singh J, Alshehri AM, Kumar D (2022). Computational study of a local fractional Tricomi equation occurring in fractal transonic flow. J. Comput. Nonlinear Dyn..

[14] Zheng B, Wen C (2013). Exact solutions for fractional partial differential equations by a new fractional sub-equation method. Adv. Differ. Equ..

[15] Wazwaz A-M (2010). Partial Differential Equations and Solitary Waves Theory.

[16] Kumar D, Singh J, Kumar S (2015). Numerical computation of fractional multi-dimensional diffusion equations by using a modified homotopy perturbation method. J. Assoc. Arab Univ. Basic Appl. Sci..

[17] Yang X-J (2016). A new integral transform method for solving steady heat-transfer problem. Therm. Sci..

[18] He J-H, El-Dib YO, Mady AA (2021). Homotopy perturbation method for the fractal toda oscillator. Fractal Fract..

[19] Liu J, Nadeem M, Habib M, Akgül A (2022). Approximate solution of nonlinear time-fractional Klein–Gordon equations using yang transform. Symmetry.

[20] Yasmin H (2022). Numerical analysis of time-fractional Whitham–Broer–Kaup equations with exponential-decay kernel. Fractal Fract..

[21] Ahmad S, Ullah A, Akgül A, De la Sen M (2021). A novel homotopy perturbation method with applications to nonlinear fractional order kdv and burger equation with exponential-decay kernel. J. Funct. Sp..

[22] Gupta PK, Singh M (2011). Homotopy perturbation method for fractional Fornberg–Whitham equation. Comput. Math. Appl..

[23] Nonlaopon K, Alsharif AM, Zidan AM, Khan A, Hamed YS, Shah R (2021). Numerical investigation of fractional-order swift-Hohenberg equations via a novel transform. Symmetry.

[24] Dehghan M, Manafian J, Saadatmandi A (2010). Solving nonlinear fractional partial differential equations using the homotopy analysis method. Numer. Methods Partial Differ. Equ. Int. J..

[25] Akbarzade M, Langari J (2011). Application of homotopy perturbation method and variational iteration method to three dimensional diffusion problem. Int. J. Math. Anal..

[26] Prakash A, Kumar M (2017). Numerical method for solving time-fractional multi-dimensional diffusion equations. Int. J. Comput. Sci. Math..

[27] He J-H, Latifizadeh H (2020). A general numerical algorithm for nonlinear differential equations by the variational iteration method. Int. J. Numer. Methods Heat Fluid Flow.

[28] Nadeem M, Li F, Ahmad H (2019). Modified Laplace variational iteration method for solving fourth-order parabolic partial differential equation with variable coefficients. Comput. Math. Appl..

[29] Dubey VP, Kumar D, Alshehri HM, Singh J, Baleanu D (2022). Generalized invexity and duality in multiobjective variational problems involving non-singular fractional derivative. Open Phys..

[30] Dubey VP, Singh J, Dubey S, Kumar D (2023). Some integral transform results for Hilfer–Prabhakar fractional derivative and analysis of free-electron laser equation. Iran. J. Sci..

